# Progress in L2-Based Prophylactic Vaccine Development for Protection against Diverse Human Papillomavirus Genotypes and Associated Diseases

**DOI:** 10.3390/vaccines8040568

**Published:** 2020-10-01

**Authors:** Pola Olczak, Richard B.S. Roden

**Affiliations:** 1 Department of Pathology, The Johns Hopkins School of Medicine, Baltimore, MD 21287, USA; polczak2@jhu.edu; 2 Department of Oncology, The Johns Hopkins School of Medicine, Baltimore, MD 21287, USA; 3 Department of Gynecology and Obstetrics, The Johns Hopkins School of Medicine, Baltimore, MD 21287, USA

**Keywords:** HPV, Human papillomavirus, vaccine, cross-protection, neutralization, papillomavirus, minor capsid protein, L2, cervical cancer, cervical intraepithelial neoplasia (CIN), anogenital cancers, skin cancer, warts

## Abstract

The human papillomaviruses (HPVs) are a family of small DNA tumor viruses including over 200 genotypes classified by phylogeny into several genera. Different genera of HPVs cause ano-genital and oropharyngeal cancers, skin cancers, as well as benign diseases including skin and genital warts. Licensed vaccines composed of L1 virus-like particles (VLPs) confer protection generally restricted to the ≤9 HPV types targeted. Here, we examine approaches aimed at broadening the protection against diverse HPV types by targeting conserved epitopes of the minor capsid protein, L2. Compared to L1 VLP, L2 is less immunogenic. However, with appropriate presentation to the immune system, L2 can elicit durable, broadly cross-neutralizing antibody responses and protection against skin and genital challenge with diverse HPV types. Such approaches to enhance the strength and breadth of the humoral response include the display of L2 peptides on VLPs or viral capsids, bacteria, thioredoxin and other platforms for multimerization. Neither L2 nor L1 vaccinations elicit a therapeutic response. However, fusion of L2 with early viral antigens has the potential to elicit both prophylactic and therapeutic immunity. This review of cross-protective HPV vaccines based on L2 is timely as several candidates have recently entered early-phase clinical trials.

## 1. Introduction

### 1.1. The Diversity of Human Papillomaviruses and the Diseases They Cause

Papillomaviruses are small DNA tumor viruses with a range of malignant potential; most infections are benign and self-limiting but a ‘high risk’ group of HPVs are the etiologic agents of 4.5% of cancers worldwide [1]. All papillomaviruses are 60 nm diameter non-enveloped T = 7*d* icosahedral viruses with a double-stranded, covalently closed and histone-bound circular DNA genome [2]. The *papillomaviridae* encompasses a very broad family of viruses that evolved to propagate in many species, including mammals and birds, but with a highly restricted host range, e.g., human papillomaviruses (HPV) only replicate in humans. Papillomaviruses also demonstrate a strict tissue tropism in that they only propagate in epithelia, and generally show a preference for either mucosa or cutaneous skin. Currently there are 226 known HPV genotypes catalogued in the Papillomavirus Episteme [3] with many more waiting to be recognized (https://pave.niaid.nih.gov/) [4]. Based upon their nucleotide sequences, the known HPVs have been classified into 5 genera termed alpha, beta, gamma, mu and nu. Species within the same genus are 60–70% similar, while novel HPV types have <90% similarity to any other HPV type [5].

**Figure 1:**
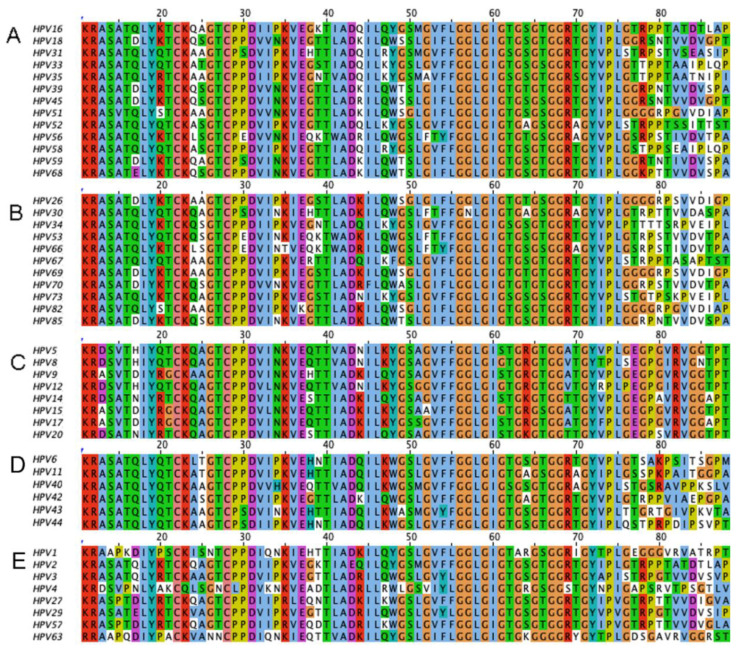
ClustalX sequence comparison of the highly conserved protective 11–88 epitope of HPV L2 minor capsid protein within groups associated with differing diseases and oncogenicity. The alignment was generated using Jalview 2.11.1.0. (http://www.jalview.org/). The default color scheme for ClustalX was used. Each of the residues is assigned a color if the threshold percentage of particular amino acids in the column was met. The following colors are assigned to the categories of amino acids: blue (hydrophobic), red (positive charge), magenta (negative charge), green (polar), pink (cysteines), orange (glycines), yellow (prolines), cyan (aromatic), white (unconserved). The aligned sequences start at the furin cleavage site conserved at the amino terminus of all L2 proteins including (**A**) High-risk alpha HPV types, (**B**) Intermediate-risk alpha HPV, (**C**) EV beta types, (**D**) Genital wart-relevant alpha types, (**E**) Benign skin wart types.

The HPV genotypes of genus alpha predominantly infect the genital tract, but other mucosa including the anal and oral cavity are also susceptible. These HPVs are highly transmissible via sexual contact and as many as 60% of people who had sexual contact with an HPV infected person will acquire the virus [6]. These alpha HPVs demonstrate a spectrum of malignant potential. The ‘low risk’ types in this genus, e.g., HPV6 and HPV11, induce benign skin warts as well as laryngeal papillomas that very rarely progress to cancer, as in the slow growing Buschke Lowenstein tumors or lung cancers, respectively. However, even these benign papillomas are associated with significant morbidity and healthcare costs. The International Agency for Research on Cancer (IARC) has identified a baker’s dozen of ‘high risk’ types, HPV16, 18, 31, 33, 35, 39, 45, 51, 52, 56, 58, 59 and 68, as carcinogenic or probably carcinogenic (Groups 1 and 2A, Figure 1A–C) detected in ~97% of cervical cancers [7]. Of these, HPV16 and HPV18 are the most impactful as they contribute to ~50% and 20% each of cases of cervical cancers worldwide [8], whereas HPV16 causes a greater fraction (>80%) in cancer types such as other anogenital and head and neck cancers [9,10]. An additional intermediate risk group (Group 2B, possibly carcinogenic, Figure 1B) appear to drive at least 2% of cases of cervical cancer comprising HPV26, 30, 34, 53, 66, 67, 69, 70, 73 and 82 [11]. Cervical cancer is the fourth most common cancer occurring in women in the world [12], of which 99% is HPV-driven [13]. The global impact of HPV-associated cancers at other sites is lower because they are less common and the attributable fraction is lower and they are primarily driven by HPV16 [14].

HPVs within the beta, gamma, mu and nu genera are mainly associated with benign infections of the squamous epithelia of cutaneous skin [15]. The gamma, mu and nu HPVs cause common warts, plantar warts and pigmented warts which typically resolve within a few months or are subclinical, but can be recalcitrant and disfiguring and require repeated destructive treatments, especially in immunocompromised patients [16].

Beta HPVs typically produce only subclinical infections or flat warts. However, they are the subject of considerable interest as mounting evidence suggests the role of a subset of types as a co-carcinogen with UV light in the development of cutaneous squamous cell carcinoma (CSCC) [17,18]. This is important because of the potential to vaccinate against the relevant beta HPV to prevent CSCC, although the currently licensed HPV vaccines do not target these types [19,20]. A causal association between beta HPV and CSCC remains controversial, but vaccination against beta HPV remains perhaps the best opportunity to directly test this hypothesis [21].

The first evidence supporting beta types as causative factors in CSCC emerged from the identification of the genodermatosis epidermodysplasia verruciformis (EV). Patients with EV frequently develop persistent flat wart-like cutaneous lesions in childhood, and they often develop CSCC later in life in sun-exposed areas. Auto/hetero-inoculation experiments linked these lesions to beta HPV infection [22], and recent genetic studies identify the role of the host’s CIB1-EVER1-EVER2 complex in intrinsic resistance to beta HPV infection [23]. While the most commonly detected beta HPVs in benign EV lesions are HPV5, 8, 9, 12, 14, 15, 17 and 19–25, it is HPV5 and 8 that are detected in 90% of CSCC of EV patients [24]. This link between beta HPV and CSCC also appears to operate in immunocompromised patients including organ transplant recipients and AIDS patients, but is controversial for the general population [25]. HPV beta types accumulate in the aging general population but to a lower extent. It is estimated that in 90% of cases, the cutaneous infections clear within 0.5–2 years [26,27]. Multiple factors including smoking or being immunocompromised contribute to slower clearance [28].

### 1.2. The Papillomavirus Genome and Proteins

The genome structure of the *papillomaviridae* is well-conserved and the 8 genes are expressed in a spatially and temporally controlled manner within squamous epithelia. There are six early genes E1, E2, E4, E5, E6 and E7 and two late genes L1 and L2, as well as a long control region (LCR) that serves as the origin of replication and to control transcription [3]. E1 and E2 have DNA-binding capability and support viral replication. E5, E6 and E7 help drive the host cell to proliferate and E4 promotes the release of virus particles from the keratinocytes by collapsing keratin filaments [2]. Interestingly, E5 is not expressed in the HPV Beta types [23].

L1 and L2 encode the major and minor capsid proteins, respectively. Both L1 and L2 are expressed in the granular layer of epithelium, but they are not detectable in the basal epithelia that harbor the infection. Each HPV capsid is composed of 72 pentamers of L1, also known as capsomer(e)s. The capsomers associate to form a virion via disulfide bridges, i.e., 360 L1 proteins per virion. Additionally, there are estimated to be 12–72 L2 proteins per virion [29], but the exact location and topology of L2 remain to be resolved. Recombinant expression of L1 in diverse systems results in its self-assembly into empty capsids, called virus-like particles (VLPs).

### 1.3. Current L1-Based HPV Vaccines and Remaining Challenges

Animal challenge studies demonstrated that vaccination with L1 VLPs provided robust, but type-restricted protection. However, L1 VLP vaccination did not impact pre-existing infection, likely because L1 is not expressed by infected basal epithelial cells. Currently, there are three licensed vaccines against Human Papillomavirus, all composed of L1-only VLPs derived from the most medically significant genotypes (Cervarix^®^ (GlaxoSmithKline Biologicals Rixensart, Belgium) and Gardasil^®^ and Gardasil 9 (Merck and Co. Inc, Whitehouse Station, NJ, USA)). Cervarix^®^ is composed of HPV16 and 18 L1 VLPs produced in insect cells and formulated with alum and the TLR4 agonist monophosphoryl lipid A (ASO4 adjuvant system). In Gardasil, the aluminum hydroxyphosphate sulfate-adjuvanted VLP are expressed in yeast and derived from L1 of HPV 6, 11, 16 and 18 for the quadrivalent vaccine, and for the predominant nonavalent vaccine HPV31, 33, 45, 52, 58, as well. They induce high and durable titers of neutralizing antibodies as the structure of the particles allows for repetitive epitope display and favors hyperactivation of B cell receptors, and they are likely protective after only a single dose. Passive transfer studies in animal models suggest that these neutralizing antibodies are central to protection, likely acting in exudates at microlesions in the epithelium that are necessary for the viral inoculum to reach and infect the basal epithelia.

Although highly efficacious and safe, these L1 VLP vaccines are limited to protection (i.e., not therapeutic) and target a limited set of mucosal HPV types, albeit the most common in cervical cancer (and genital warts for Gardasil). Therapeutic vaccines aimed at treating pre-existing infections and disease generally target early antigens, primarily E6 and E7, as they are obligately expressed in infected cells and cancer, and are being developed to help those suffering HPV-related disease. It is clear that prophylaxis against a broader range of medically relevant HPV types is desirable for: (1) protection against all carcinogenic and probably carcinogenic alpha HPV that together cause >99% of cervical cancer and eliminate the need and costs for cervical screening, (2) protection against the morbidity and treatment costs associated with benign HPV diseases beyond genital warts, (3) protection against CSCC associated with beta HPV, especially in immunocompromised patients. Unfortunately, the number of different HPV genotypes that cause these diseases is large, rendering production of a sufficiently highly multivalent L1 VLP vaccine impractical, so development of a single cross-protective antigen is of interest as an alternative. For this, the field has generally focused on the minor capsid antigen, L2 (Graphic Abstract).

### 1.4. L2 as a Broadly Protective Vaccine Antigen

The L2 protein is composed of ~500 amino acids and it is required for infection. L2, by itself, does not form the VLPs, but when co-expressed with L1, it co-assembles into capsids and facilitates the encapsidation of the viral genome [30]. Most of the L2 protein is buried within the capsid, but portions are exposed on the capsid surface [31]. L2 was first recognized as a potential antigen for use in prophylactic immunization in the early 1990s. Foundational studies in cattle (bovine papillomavirus, BPV, types 1, 2 and 4) and rabbit (cottontail rabbit papillomavirus, CRPV) challenge models demonstrated that vaccination with L2 produced recombinantly in bacteria can provide durable protection, but it does not impact pre-existing infection [32,33,34,35,36]. Passive transfer studies suggest that neutralizing antibodies confer immunity [37,38]. However, the serum titers of neutralizing antibodies measured in vitro induced by L2 vaccination are substantially lower than achieved with L1 VLP. Perhaps as a result, the development of L2 vaccines languished.

Interest in L2-based vaccination was re-kindled by two observations. First, vaccination with N terminus of the L2 protein elicits both neutralizing antibodies and protective immunity [32]. Second, because the protective epitopes at the N terminus of L2 demonstrated robust sequence conservation (Figure 1), vaccination with this region induced broadly neutralizing antibodies against both mucosal and cutaneous types [39,40] and cross-protection against highly divergent papillomaviruses [41]. These observations were not restricted to animal studies. Vaccination of patients with full-length HPV16 L2 fusion protein even without adjuvant elicited cross-neutralizing antibodies [42]. This suggested the hypothesis that a single L2-based immunogen might provide broad protection and overcome or complement the type-restricted immunity elicited by L1 VLP.

**Figure 2:**
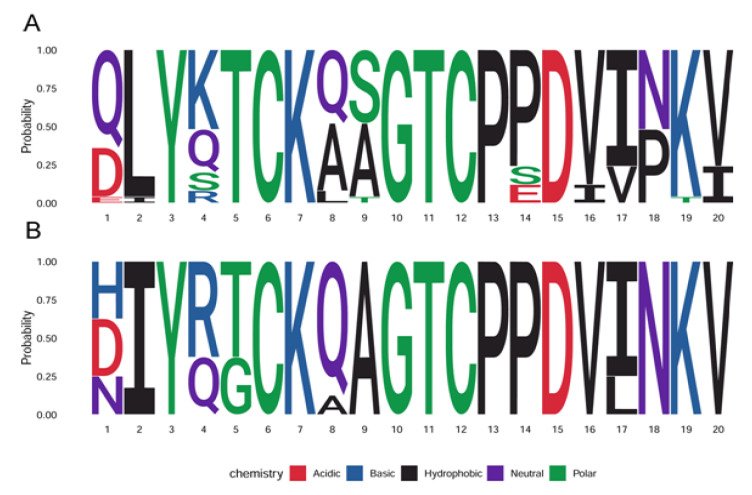
Designing consensus sequences within a conserved protective epitope of L2 of alpha and beta HPV genotypes. Plot of the probability of listed amino acids at given position within the RG-1 sequence (i.e., defined by HPV16 L2 17–36) of L2 of medically significant HPV types. (**A**) Oncogenic or probably oncogenic alpha types (HPV16, 18, 26, 30, 31, 33, 34, 35, 39, 45, 51, 52, 53, 56, 58, 59, 66, 67, 68, 69, 70, 73, 82 and 85). (**B**) EV-associated HPV beta types (HPV 5, 8, 9, 12, 14, 15, 17, 20). The figures were generated using ggseqlogo package in R studio.

This hypothesis triggered renewed focus on defining the cross-protective epitopes displayed by L2 and how best to present them to the immune system to trigger a robust and durable immunity against the plethora of HPV types with significant medical impact (summarized in Graphic Abstract). These epitopes within L2 were defined by generating cross-neutralizing antibodies or by peptide vaccination. Examples include the identification of the RG1 cross-neutralizing monoclonal antibody which recognizes amino acids 17–36 of L2 [37,43,44] (Figure 2), or Mab5 and Mab13 which recognize residues 69–81 and 108–120 respectively [45,46]. Interestingly, while natural infection or vaccination with virions or L1 + L2 VLP induces significant L1-specific antibody responses, they result in little or no L2-specific neutralizing antibody responses, as L2-neutralizing epitopes are buried and exposed only transiently during infection [47]. The remainder of the review concerns efforts to enhance the presentation of L2 within an immunogen to induce durable and broad immunity elicited by robust neutralizing antibody levels (Table 1).

### 1.5. How You Measure Neutralizing Antibodies Matters

Neutralizing antibody titers are an important correlate of protection for prophylactic vaccines [88]. Assay of the serum antibody titer after L2 vaccination using ELISA suggests a robust response. Likewise, passive transfer of immune sera confers strong protection. However, measurement of in vitro neutralization titer using native virions or pseudovirions (infectious L1/L2 particles carrying a reporter construct) suggests a weak antibody response despite complete protection to challenge [38]. Day et al. recently showed that the typical in vitro neutralization assay format poorly detects L2-specific neutralizing antibodies as compared to L1-specific antibodies. This likely reflects differences in the in vitro infectious process (e.g., of 293TT target cells) and that in vivo on the basement membrane at the site of epithelial damage. A conformational change is induced by L1-mediated capsid binding to the heparan sulfate proteoglycans of the basement membrane which exposes the amino acid terminal of L2 of the capsid surface, where it can be cleaved by furin, a pro-convertase enzyme critical to infection [89,90,91]. Cleavage of L2′s amino terminus by furin promotes infection of the keratinocyte but renders the virion susceptible to L2-specific neutralizing antibodies [92,93,94]. Because this process is rapid in tissue culture but slow in vivo, the sensitivity for measurement of L2-specific neutralizing antibodies can be substantially improved by using furin pre-cleaved virus for in vitro assays [89,95,96]. Unfortunately, this is a recent observation, and most published studies utilize the first generation neutralization assays that sensitivity to L2-specific neutralizing antibodies, an important caveat in interpreting their findings.

### 1.6. L2 Vaccines Based on (Poly)peptides

Since vaccination of animals and patients with full-length L2 induced low titers of cross-neutralizing epitopes [42], one possibility was that the antibody response was focused on non-neutralizing regions of L2 that were never exposed on the capsid surface. This might be addressed by focusing the response on the relevant regions by vaccinating with only the protective epitopes. Kawana et al. identified HPV16 L2 residues 108–120 as a cross-protective epitope and showed it was immunogenic in mice via intranasal administration in the absence of adjuvant [50]. When it was similarly administered to patients the HPV16 L2 108–120 peptide induced cross-neutralizing antibodies in 4/5 of patients in the high dose (0.5 mg at weeks 0, 4 and 12) group, but 0/5 at 0.1 mg [51]. This important study suggested the need for an adjuvant and potentially inclusion of T helper epitopes and/or using a longer sequence, which might also confer the advantage of increasing cross-reactivity to more HPV genotypes in addition to providing T helper epitope(s). The immunogenicity of peptide vaccines has been increased by chemical coupling of protective L2 epitopes to the surface amino acids of a protein carrier, typically keyhole limpet hemocyanogen (KLH) which provides a source of T helper epitopes and potentially irregular multimerization tethered at one end only [35,37,56,57]. These vaccines are able to elicit a protective immunity when administered to animal models with adjuvant.

Longer polypeptides can contain multiple protective epitopes and T helper epitopes. Vaccination with full-length L2 protein recombinantly expressed and purified from bacteria induces low levels of neutralizing antibodies and is protective. Cattle vaccinated with the N-terminal 11–200 polypeptide of the L2 of bovine papillomavirus type 4 (BPV4) showed stronger protection against BPV4 challenge and higher serum neutralizing antibody titers than animals vaccinated with the remainder or full-length L2 protein [52]. Vaccination of rabbits with residues 1–88 of bovine papillomavirus type 1 (BPV1) L2 elicited significantly higher titers than full-length protein, and the antisera very broadly neutralized diverse papillomavirus types [40]. Rabbits immunized with L2 polypeptides comprising amino acids 11–88 or 11–200 of cotton rabbit papillomavirus (CRPV), BPV1 and HPV16L2 showed protection against skin challenge with CRPV but also against rabbit oral papillomavirus (ROPV) [41]. L2 polypeptide vaccination did not protect against CRPV DNA challenge, confirming a lack of therapeutic activity. These studies supported the use of a longer L2 polypeptide, but the titers of neutralizing antibodies were much lower than for L1 VLP.

One reason for the high immunogenicity of L1 VLP is repetitive display of the protective epitopes. Jagu et al. explored whether creating concatemers of the L2 protective epitopes, from 11–200, 11–88 and 17–36 would likely increase immunogenicity, and whether the number of repeats mattered by testing 3, 5, 8, 15 and up to 22 repeats [53]. To increase the breadth of the cross-protective immune response, they used an L2 derived from a different HPV type in each repeat. A fusion of L2 11–88 of five or eight different HPV types proved the most effective, and vaccination with this approach using only alum adjuvant provided robust protection against all HPV genotypes tested in the mouse and rabbit challenge models [53,54]. Passive transfer studies indicate that protection was mediated by neutralizing antibodies [54]. This approach is currently being advanced toward the clinic by Bravovax.

Another approach to increase the immunogenicity of multimers of protective L2 peptides is by insertion into a heterologous protein scaffold that itself activates T cell proliferation [58], such as the small redox protein, thioredoxin (Trx) [97]. L2 peptide fusion to thioredoxin showed increased immunogenicity when compared to L2 linear peptides and showed that multimers of the epitopes are more immunogenic than the monomers [58]. Peptide composed of amino acids 20–38 of HPV16 L2 protein fused to Trx produced antibodies that only elicited an immune response against the HPV16 L2 antigen and did not cross neutralize other tested HPV types. Inclusion of L2 sequences from additional HPV types (HPV31 and 51) fused to a single Trx molecule elicited high response to all three types that were covered in the vaccine. The response to this immunization in both mice and guinea pigs was high and robust [59]. This approach has been improved by generating a fusion of residues 20–38 of 8 mucosal HPV types and conjugating it to a thermostable thioredoxin derived from a thermophile archaebacterium [60,61]. The immunogenicity of this vaccine was further improved by the addition of the OVX313 heptamerization domain. The addition of the heptamerization domain also enhanced the T-helper response [62]. Martin Muller (DKFZ) is advancing their product for clinical testing.

### 1.7. L2 Epitopes Fused to TLR Ligands

The above vaccines are formulated by mixing with a conventional adjuvant prior to administration, but improved immunogenicity might be achieved by direct conjugation of L2 to a defined adjuvant, such as a toll-like receptor (TLR) agonist. HPV16 L2 RG1 peptide fused with a broadly recognized T helper epitope (P25) and toll-like receptor 2 (TLR2) ligand dipalmitoyl-S glycerin cysteine (P2C) generated a potent response far superior to the linear epitope. Vaccinated mice were protected against challenge with HPV16 and HPV45. Sera from vaccinated mice neutralized other papillomavirus types, including HPV5 and BPV1 [63]. This vaccine was generated by direct chemical synthesis, but it requires inclusion of T helper epitopes recognized by the broad and HLA diverse human population. To this end, Zhang et al. generated a lipidated triple RG1 epitope multimer fusion protein fused with a single-chain antibody fragment targeting human FcγRI. Vaccination with this protein purified from bacteria elicited cross-neutralizing antibodies against a broad spectrum of HPV types, but surprisingly still needed additional adjuvant despite carrying a TLR2 ligand [64].

Flagellin is a TLR5 ligand that was tested as a direct fusion to multimer of L2 protective epitopes from various types (HPV6, 18, 31, 39, 52) and was potently immunogenic at low doses without adjuvant [65]. Importantly, it conferred broad protection against diverse HPV genotypes in rabbits and immunity was retained over a one-year period post vaccination [55]. Likewise, concatemers build out of RG1 epitopes of HPV18, 33, 58, 59 and amino acids 11–88 of HPV16 L2 fused to flagellin induced cross-neutralizing antibodies in mucosal secretions and protection from vaginal challenge when administered subcutaneously or intranasally to mice [66].

### 1.8. L2 Displayed on Bacteria

Bacteria express several TLR ligands that could adjuvant an L2 vaccine response, and the use of lactobacillus as a vector for mucosal vaccines can promote strong responses [98]. Lactobacilli-based recombinant vaccines are considered safe as compared to most other live-vaccine vehicles and can be produced at a low cost. When *Lactobacillus casei* (*L. casei*)-expressing residues 1–224 of HPV16 L2 fused to the poly-γ-glutamic acid synthetase A (pgsA) surface protein were administered to Balb/c mice, they elicited neutralizing antibodies against HPV16 and other mucosal types including HPV18, 45, 58 and BPV1, but no protection studies were performed [67].

As an alternative approach to provide surface display for L2 epitopes in the presence of TLR ligands, the outer membrane proteins (OMP) of a gram-negative bacterium that span membranes multiple times can be utilized, e.g., the trimeric OmpF in *E. coli* form a pore in outer membranes. The L2 antigen inserted in OmpF was expressed in the *E. coli* and stably presented on the outer membrane, but the immunogenicity was not tested [68].

### 1.9. L2 Epitopes Displayed on Papillomavirus L1 VLP

The L1 VLP vaccines induce high-titer antibodies even without adjuvant, and these responses are durable. This is believed to reflect the high density and close spacing of neutralizing epitopes that each particle displays (i.e., 360 copies on a 60 nm diameter particle) because this efficiently cross-links and activates B cell receptors, as well as promoting opsonization and uptake by antigen-presenting cells [99]. By contrast, L2 is much less immunogenic because it does not form a particle and, when expressed alone, is a monomeric antigen. Therefore, many groups have attempted to increase the immunogenicity of L2 by displaying it in the immunodominant neutralizing epitope(s) of a VLP. This can be challenging because the assembly and structure of VLP often are compromised by insertion of new sequences, and this is generally more problematic with larger insertions. Therefore, typically only small conserved neutralizing epitopes of L2 are used for insertion, e.g., residues 17–36, 65–81 or 108–120. Selection of the insertion site can be guided by known VLP structures, if available, to identify the immunodominant surface epitopes in the carrier capsid antigen. However, this nevertheless remains quite empiric because most such epitopes are conformational rather than linear so it is hard to predict the impact of insertion on the structure and immunogenicity of the recombinant.

HPV L1 is a logical carrier for the L2 epitope. Unfortunately, the cross-protective L2 epitopes are mostly buried in native virions or L1/L2 VLP and therefore do not effectively trigger an L2 response, which is why only L1 is used in the licensed VLP vaccines. Several groups began to identify potential insertion sites in L1 for conserved L2 protective epitopes, including residues 17–36, 65–81 or 108–120, by focusing on the surface loops [100,101,102]

The DE loop of the L1-based VLP is particularly useful for such display, and it allows for the presentation of an antigen on the surface of the particles. One promising technology is a chimeric VLP that has an RG1 epitope derived from HPV16 displayed on the HPV16 L1 VLP surface [103]. Vaccination with this HPV16L1-16RG1 VLP chimeric vaccine elicits serum antibody responses that protect against a wide range of high-risk HPV types via passive transfer [70]. Importantly, this insertion does not appear to compromise L1-specific immunity [71], and insertions of L2 epitopes from other HPV types is also possible [69,72], or even consensus-derived sequences for alpha and beta HPV-derived L2 ‘RG1 epitope’ which are slightly different as shown in Figure 2. The HPV16L1-16RG1 VLP vaccine is being prepared for clinical studies with support from the NCI PREVENT and SPORE programs, and commercially by Pathovax LLC.

It is possible to use other L1 for display, including HPV18 [104] or BPV1 [73]. For example, a similar vaccine was developed in the HPV18 L1 backbone displaying an HPV45-derived RG1 epitope [105]. While the HPV16-derived RG1 epitope is well-conserved among the hrHPV (alpha) genotypes, the homology and cross-reactivity of antisera is lower for the cutaneous beta HPV types. One approach is to display an RG1 epitope derived from such a representative beta HPV, e.g., the HPV17 RG1 sequence was displayed on the HPV16L1 DE surface loop. Immunization of rabbits with these chimeric VLP produced robust antibody titers against HPV16 L1 VLP, as well as cross neutralizing responses against a variety of HPV beta types, including 5, 8, 20, 24, 36, 23, 80, 49, 92, 96. When in vivo cross-protection was evaluated by passive serum transfer in a murine PsV challenge model, the immune sera to HPV16L1–17RG1 VLP (cross-) protected against beta HPV5/20/24/38/96/16 (but not type 76) [74]. While most studies were performed using baculovirus-based expression, other recombinant systems such as plants [101], yeast [106,107] and possibly even bacteria [108] can also be used.

However, while displaying a conserved L2 epitope on L1 VLPs elicits broad protection via induction of antibodies neutralizing for multiple HPV types that are not included in the vaccine, challenges remain. First, the L2-reactive titers, although associated with cross-protection, are low compared to the L1 VLP-specific responses. This raises concerns about the longevity of cross-protection induced by these HPV16L1–16RG1 VLP, although recent studies in rabbits show broad protection is maintained over 1 year using alum-formulated HPV16L1–16RG1 VLP [109]. Secondly, the conservation of the RG1 epitope is imperfect, and particularly for the beta HPV, suggesting that use of a consensus epitope (Figure 2), or adding a construct with a second different epitope inserted at a different site, and use of a stronger adjuvant like AS04, might be used to cover these types more comprehensively [104].

The HPV16L1–16RG1 VLP (or similar such L2 chimeric construct) might be combined with a conventional multimeric L1 VLP vaccine to help fill in the gaps in coverage of hrHPV genotypes not directly targeted [71,104]. Likewise, no interference with L1 responses was seen when mixing L1 VLP or capsomers with L2 multimer [110], or when fusing to the C-terminus of L1 in a capsomer vaccine [111].

### 1.10. HPV L2 Epitopes Displayed on Other Eukaryotic Viruses and Their VLP

VLPs generated from the major capsid antigens of many other viruses have also been tested as carriers for surface presentation of L2 epitopes, including hepatitis B core virus-like particles displaying HPV16 L2 14–122 which induced HPV16 neutralizing antibodies [75], as well as potyvirus-like particles [76] and grapevine fanleaf virus (GFLV) VLPs [77] which were not characterized for immunogenicity.

Adeno-associated viruses (AAV) are small, single-stranded DNA paroviruses used in many human gene therapy studies. Their Rep genes code for proteins involved in viral replication and packaging while Cap genes code for structural proteins [112]. When one of the AAV structural proteins, VP3, is expressed along with assembly activating protein (AAP), VLPs can be formed (AAVLP). Recombinant AAVLPs with insertions of both HPV16 and HPV31 L2 residues 17–36 were prepared, as their sequences differ slightly between the two types. Incorporation of both in the AAVLP did not interfere with the capsid assembly and vaccination with the AAVLP (HPV16/31L2) induced broadly neutralizing antibodies against various types, including HPV18, 45, 52, 58 [78]. The use of adjuvant was necessary in mice both for the induction of L2-specific neutralizing antibody and protection against vaginal challenge with HPV16. Co-formulation with alum elicited protection >3 months, but antibody titers were increased by addition of MPL adjuvants. To determine the breadth of immunity, rabbits were immunized three times with AAVLP (HPV16/31L2) either alone, formulated with alum ± MPL, or RIBI adjuvants, and the animals were concurrently challenged with quasivirions with the seven most common oncogenic HPV types or cottontail rabbit papillomavirus (CRPV). Strong protection against all HPV types was observed at both 6 and 12 months post immunization, including robust protection in rabbits receiving the vaccine without adjuvant. Based upon these findings, 2A Pharma has performed a placebo-controlled phase I study assessing safety, tolerability and immunogenicity of the AAVLP (HPV16/31L2) vaccine in healthy adult male and female subjects (NCT03929172). This is the most advanced effort to develop an L2-based preventive HPV vaccine, and the results of this trial are eagerly awaited.

Replication-incompetent adenovirus vectors have been widely tested as vaccines in humans, as they have proven safe and potent. Adenovirus type 5 (Ad5) hexon’s immunodominant neutralizing epitopes have been mapped to particular hypervariable regions (HVR). An L2 epitope comprising of residues 12–41 was inserted or substituted into HVR1 or HVR5 within the context of the virus by homologous recombination. The inclusion of the HPV L2 fragments did not affect the ability of the virus to replicate in 293 cells. Vaccination of mice, in which Adenovirus 5 cannot replicate, induced low neutralizing antibody titers against the L2 epitope as compared to the native virus, although the L2-specific antibody titers could be increased by an addition of adjuvants such as alum and monophosphoryl lipid A [79]. This suggests that not all insertion sites work well, and that predicting the best location to display L2 within a heterologous virus capsid is hard to predict and must be test empirically. Interestingly, these L2-displaying adenovirus 5 constructs would be replication-competent in humans, which would likely enhance the L2-specific antibody responses, although the impact of pre-existing Adenovirus 5 immunity might blunt them.

Vujadinovic et al. chose to display concatemers of L2 aa 17–36 epitopes from multiple HPV types at the C-terminus of protein IX of a rare human adenovirus type 35 (HAdV35) to allow incorporation of larger regions and to avoid issues of pre-existing immunity, respectively. This approach also leaves one end of the L2 unconstrained, perhaps better mimicking furin-cleaved L2 in the infectious intermediate [113]. Insertion did not compromise replication in an E1 complementing cell line, and the recombinants were stable. Upon vaccinating mice without the use of adjuvant, robust antibody responses were detected by ELISA, as well as neutralization of diverse hrHPV types, although protection from experimental challenge was not examined [80].

Instead of genetic insertion, another approach to coupling is the biotinylation of particles [114], in this case Tobacco Mosaic Virus (TMV), and coating them with L2 expressed as a fusion with streptavidin [87]. This latter approach allows for the conjugation of larger polypeptides, in this case residues 61–171 of L2. However, chemical coupling is more complex to control during clinical grade manufacture and therefore most groups have focused on genetic insertion. Indeed, the same group inserted L2 residues 94–122 of cottontail rabbit papillomavirus (CRPV) or rabbit oral papillomavirus (ROPV) into position 155 of TMV’s U1 coat protein, and showed that vaccination of rabbits with the CRPV L2-recombinant TMV provided robust protection against experimental CRPV challenge, but not with ROPV L2-recombinant TMV, reflecting quite divergent sequences in this region [81].

### 1.11. L2 Epitopes Displayed on Bacteriophage and Their VLP

Antigen display can also be accomplished either by genetic insertion or chemical conjugation of L2 peptides into the capsid surface of bacteriophage like Qβ, an ssRNA bacteriophage which grows in *E. coli*, or VLPs derived from the phage PP7 [82], an ssRNA bacteriophage of *Pseudomonas aeruginosa*. In this study, the breadth of cross-reactivity of the L2 residues 65–85 was broadened by using a consensus sequence derived from hrHPV types to couple to the particles.

The insertion of HPV16 L2 17–36 epitope into a surface loop (AB) displayed on a PP7 bacteriophage coat protein dimer allowed for proper VLP assembly without discernable changes to the PP7 morphology. Immunization of mice with these VLPs induced robust L2-specific antibody responses that neutralized and protected mice from vaginal challenge with either HPV16 or HPV45 pseudovirus [115]. Intravaginal vaccination with PP7-L2 VLP was also protective, but not as effective as intramuscular vaccination [83]. However, although some cross-protection occurred, for example against HPV45, it was not strong [115]. PP7 VLPs were able to display the amino acids 17–31 (or equivalent) of L2 of multiple HPV types, and vaccination of mice with a mix of 8 of these PPV-L2 VLP provided robust protection against vaginal challenge with diverse HPV pseudovirion types [84]. Vaccinated mice were strongly protected over one year post immunization, and exogenous and endogenous adjuvant (encapsidated single-stranded RNA) had minor effects on antibody titers which also lasted > 18 months [85].

Since the goal is a single cross-protective construct, an alternative approach to unconstrained display of L2 at the N-terminus of the RNA bacteriophage MS2’s coat protein, to contrast with the presumably more rigid display of L2 within the AB loop of PPV7 coat protein, was tested. Amino acids 20–29, 17–31, 14–40 and 14–65 of HPV16 L2 were each genetically inserted at the N-terminus of the single-chain dimer of MS2 coat protein, and all assembled except for the 14–65 construct, suggesting a potential insertion size limitation. The MS2 VLP displaying HPV16 L2 17–31 elicited the broadest and most robust protection against vaginal challenge with diverse HPV genotypes [86]. These particles are amenable to spray drying, which can potentially stabilize the product to facilitate global delivery without the need to maintain a cold chain [116,117,118].

Breadth of cross-protection could also be further enhanced by incorporation of two epitopes into a single hybrid particle [119], or by the display of a consensus L2 protective epitope [82], or both to achieve similar levels of protection of mice against vaginal HPV pseudovirus challenge with a single MS2–31/16L2 VLP as achieved with Gardasil 9 [120].

### 1.12. Combination of L2-Based Prophylaxis and Therapeutic HPV Vaccination

The utility of L2 and L1 VLP vaccines is restricted to prophylaxis presumably because the basal keratinocytes that harbor the viral infection do not detectably express either capsid antigen, and therefore are not targeted, whereas the infected cells in the upper epithelial layers that do express them are already dying. Therefore, therapeutic ones targeting HPVs are being developed, and are frequently designed to target the viral oncogenes, E6 and E7, since they are obligately expressed in all HPV-infected cells [121]. Other early viral antigens are sometimes targeted, including E1 and E2, but they are not typically expressed in HPV-related cancers. There have been efforts to combine both types of vaccine, and several such constructs have reached the clinic. Unfortunately, none of the trials address their ability to prevent new infections, but rather focus on triggering regression of existing HPV-related anogenital intraepithelial neoplasia (AGIN) or warts, as summarized in Table 2. However, TA-CIN, a fusion protein composed of HPV16 L2, E7 and E6, was studied in healthy volunteers. It was well-tolerated and induced a significant antibody response even without the use of adjuvant, likely reflecting its amorphous particulate nature [122]. The sera from the vaccinated patients neutralized HPV16, cross-neutralized other hrHPV [42], and conferred protection via passive transfer to naïve mice against vaginal challenge [96]. However, an adjuvant would likely be necessary to achieve durable and robust immunity [123]. The TA-GW vaccine is a fusion protein that consists of the L2 and E7 proteins of HPV6 expressed in *E. coli*, and is administered as an amorphous antigen on alum. Phase I studies in healthy volunteers [124] and a phase IIa clinical trial in 25 genital wart patients have shown immunogenicity in the form of T cell responses and serum antibodies [125]. In the phase IIa study, five patients completely cleared warts within eight weeks. Those whose warts were not cleared by eight weeks were offered conventional therapy. Recurrence of warts was not seen in any of the 13 persons whose warts cleared by vaccine alone or with conventional therapy, and the relative contributions of the T cell and antibody immunity to these observations remains to be determined. A larger trial using AS02A adjuvant failed to show benefit for HPV6 L2E7 vaccination to enhance the efficacy of treatment of genital warts, but prophylaxis was not examined [126]. Both the L2-specific antibodies and the cellular immune responses against early antigen could potentially be protective against both new infection and re-infection, although the T cell response is unlikely to be cross-type reactive beyond the very closely related HPV11.

## 2. Conclusions

Currently licensed HPV vaccines offer prophylaxis against a limited range of HPV types, albeit the most common types in anogenital cancer, with low potential of cross-reactivity. It is not feasible to generate sufficiently multivalent L1 VLP vaccine formulations to cover all the oncogenic HPV types, let alone the many other benign types that cause considerable morbidity for patients. Therefore, there remains considerable interest in using an L2-based technology to vaccinate against most, if not all disease-relevant HPV types. This breadth of protection elicited by the well-conserved amino acid sequence of the protective epitopes of L2 may be especially important in immunocompromised patients such as organ transplant recipients and HIV co-infected patients who develop disease associated with a plethora of unusual HPV types.

Although the L1 VLP vaccines produce high antibody titers, durable response and long-term protection, they do not have therapeutic potential. L2-based vaccines alone do not have therapeutic potential, but when fused with early proteins, notably the viral oncoproteins E6 and E7, they might induce both prophylactic and therapeutic immunity. This might be particularly advantageous for catch-up vaccination in older populations already exposed to HPV and thereby reduce cervical cancer rates sooner.

There are many hurdles to the development of a broadly protective L2-based vaccine, including the success of the L1 VLP vaccines. Currently, the most challenging one is ensuring the longevity of L2-based immunity given the relatively lower neutralizing antibody titers than seen for the licensed vaccines, and the likely need for more than a single vaccination. However, it is important to appreciate that the neutralization assays detect protective L2 antibodies poorly, and that passive transfer assays are more informative [43].

We have described the numerous L2-based strategies tested with a goal of achieving durable immunity, and virus-like display appears the most promising. However, immunity lasting over a year in animals has also been seen with repetitive display via L2 multimers formulated in alum or as a flagellin fusion. Furthermore, mice immunized with a single dose of MS2–16L2 VLPs were partially protected from challenge with a high dose of HPV16, one year after immunization even without the use of adjuvant [116]. Single dose immunization may not be such an advantage if the L2-based vaccine is incorporated in combination with other vaccines, e.g., hepatitis B, which have a multidose regimen. In addition, L2 vaccines can target common skin warts that afflict children, providing a rationale to incorporate them into a pediatric vaccination regimen that does not exist for the currently licensed L1 VLP vaccines.

A particularly interesting emerging area of opportunity for L2 vaccination is in prevention of cutaneous squamous cell cancer (CSCC). Despite the use of sunscreen and costly screening and intervention by dermatologists [129], there are 4000–8000 deaths from CSCC in the US every year [130]. CSCC in immunocompromised patients is associated with a plethora of beta HPVs, and this relationship was first described in EV [131,132,133]. While beta HPV infections are clinically inapparent in normal patients, in EV patients they produce disfiguring warts covering which progress to CSCC in sun-exposed areas [134]. The beta HPV are associated with CSCC in organ transplant recipients (OTR) and HIV+ patients, but the link to CSCC in aging but healthy individuals is less clear [135]. While UV exposure is the mutagen, beta HPV infection appears to act by compromising repair of UV-induced DNA damage in the skin. Beta HPV episomes and early gene expression can readily be detected in the precursor lesions, actinic keratosis, and in well-differentiated keratinizing SCC. However, unlike oncogenic alpha HPV, continued expression of beta HPV may not be required, i.e., a hit-and-run mechanism, as seen in poorly differentiated non-keratinizing SCC. No commercial vaccine, including Gardasil9, targets the beta HPVs that cause CSCC. The 50 known cutaneous beta HPVs are too many to practically target with L1 VLP vaccination. However, an L2-based vaccine that broadly protects against all oncogenic βHPV has the potential to reduce the burden of cutaneous squamous cell cancers [136].

## Figures and Tables

**Table 1:** Summary of published L2 vaccine technologies

Technology for Display	Description	Region of L2	Citations
Linear monomer, and multimers of L2 (poly)peptides	L2 protein	Full length L2 protein	[34,36,42,48,49]
	Synthetic HPV16 L2 peptide	HPV16 L2 AA 108–120 peptide	[50,51]
	BPV4 L2 polypeptide	BPV4 L2 AA 11-200 polypeptide	[52]
	BPV1 L2 polypeptide	BPV1 L2 AA 11–88 polypeptide	[40]
	L2 multimers	HPV (6, 16, 18, 31, 39, 51, 56, 73) L2 AA 11–88 or 11–200	[41,53,54,55]
L2 peptides linked to KLH	L2 peptides	Various HPV types and BPV4, L2 short peptides	[35,56,57]
Thioredoxin fused peptides	Thioredoxin conjugated concatemers	HPV16 L2 AA 1–120 (20–38; 28–42; 56–75; 64–81; 96–115; 108–120) fused to thioredoxin HPV 16, 31 and 51, AA 20–38 fused to thioredoxin	[58,59]
		HPV16 AA 20–38 × 3 fused to PfTrx HPV16 AA 20–38 × 3 fused to PfTrx (+heptamerization domain)	[60,61,62]
L2 epitopes fused to TLR ligands	L2 peptide fused to Th and P2C	HPV 16 L2 AA 17–36 fused with T helper epitope (P25) and dipalmitoyl-S glycerin cysteine (P2C)	[63]
	RG1 epitopes fused with human Fc and lipidated	HPV (multiple types) AA 17–36 fused with antibody fragment targeting human FcγRI	[64]
	L2 linear multimers fused to Flagellin	HPV-16 L2 11(AA 11–200)/L2 fusion of 5 or 8 HPV types, AA 11–88 peptides	[65]
		HPV 8, 33, 58, 59, AA 17–36 of L2 and HPV16 AA 11–88 of L2	[66]
L2 displayed on bacterial surface	HPV16 L2 displayed on *Lactobacillus casei*	HPV16 AA 1–224 of L2 fused to poly-γ-glutamic acid synthetase A (pgsA)	[67]
	L2 antigen inserted in OmpF was expressed in *Escherichia coli*	AA 17–33	[68]
Display of L2 on papillomavirus L1 VLP	Chimeric HPV16 L1-RG1 cVLP	HPV16, 18, 31, 58 AA 17–36 of L2	[69,70,71,72]
	Chimeric various HPV L1-RG1 cVLP	HPV4, 5, 17, 45, AA 17–36 or 53–72 of L2	[73,74]
HPV L2 displayed on eukaryotic viruses and their VLP	HPV16 L2 displayed on hepatitis B core virus-like particles	HPV16 L2, AA14–122 of L2	[75]
	HPV16 L2 displayed on potyvirus-like particles	HPV 16 L2 AA 108–120	[76]
	HPV16 L2 displayed on grapevine fanleaf virus (GFLV) VLPs	L2 17–31	[77]
	HPV16/31 L2 epitopes displayed on AAV2 particles	HPV16, 31 AA 17–36 of L2	[78]
	L2 displayed on human Adenovirus 5	HPV16 L2 AA 12–41	[79]
	L2 displayed on protein IX of human Adenovirus 35	Various concatamers of different HPV types, AA17–36 of L2	[80]
	CRPV or ROPV, L2 AA 94–122	CRPV or ROPV, AA 94–122 of L2	[81]
L2 epitopes displayed on bacteriophage and their VLPs	HPV L2 peptide VLP displayed on PP7	HPV1,5,6,11,16,18, 45, or HPV58, AA 65–85 of L2 HPV16 AA 17–36 of L2 17–31 (or equivalent) of L2 of multiple HPV types	[82,83,84,85]
	HPV L2 displayed on MS2 coat protein	HPV16 L2 17–31	[86]
	CRPV/ROPV L2 display on U1 of TMV	COPV L2 61-171 CRPV/ROPV L2 94-122	[87] [81]

**Table 2:** Summary of clinical studies testing vaccines that include the minor capsid protein L2

Vaccine	Specific Condition	Phase	Technology Used	Citations
**Vaccines with Prophylactic and Therapeutic Potential**				
TA-CIN	Healthy volunteers High-grade AGIN HPV16+ VIN/High-grade AGIN HPV 16+ Cervical Cancer	Phase I Phase II Phase I	Fusion protein HPV16 L2-E7-E6	[122,127,128] Cantab Pharmaceuticals/Xenova NCT02405221
TA-GW	Healthy volunteers/Genital warts	Phase I Phase IIa	Fusion protein HPV6 L2E7	[124] Cantab Pharmaceuticals/Xenova
pNGVL4a-CRTE6E7L2 DNA	HPV16+ CIN2/3	Phase I	pNGVL4aCRTE6E7L2 HPV DNA Vaccine + electroporation	NCT04131413
PVX-6	HPV16+ ASC-US, ASC-H, LSIL	Phase I	pNGVL4aCRTE6E7L2 DNA i.m. vaccination twice and single IM TA-CIN	NCT03913117
PVX-2	HPV16+ ASC-US, ASC-H, LSIL	Phase II	pNGVL4aSig/E7(detox)/HSP70 DNA i.m. vaccination twice and single IM TA-CIN	NCT03911076
**Vaccines with Only Prophylactic Potential**				
HPV16 L2 AA 108–120 peptide	Healthy volunteers	Completed	Synthetic peptide consisting of the AA 108–120 of HPV16 L2	[51]
αHPV L2 multimers	Oncogenic and cutaneous papillomavirus infections	In preparation	L2 11–88 of five or eight different αHPV	Bravovax
Thioredoxin- conjugated L2	Oncogenic and cutaneous papillomavirus infections	In preparation	Heptamerized L2 8-mer thioredoxin single-peptide antigen	DKFZ
HPV16L1–16RG1 VLP	Oncogenic and cutaneous papillomavirus infections	In preparation	RG1 display of on L1 VLP	NCI PREVENT and SPORE Pathovax LLC
AAVLP-HPV	Papillomavirus infections	Phase I	HPV16 and 31 RG1 insertion on AAVLP	2A Pharma AB NCT03929172
